# Two-Dimensional Magnetic Orientation of Steel Fibers in Large Slab Elements Made of HPFRC Using an Industrial Robot

**DOI:** 10.3390/ma19010125

**Published:** 2025-12-30

**Authors:** Kristýna Carrera, Petr Konrád, Karel Künzel, Přemysl Kheml, Radoslav Sovják, Michal Mára, Jindřich Fornůsek, Petr Konvalinka

**Affiliations:** 1 Experimental Centre, Faculty of Civil Engineering, Czech Technical University in Prague, Thákurova 7, 166 29 Prague, Czech Republic; kristyna.carrera@fsv.cvut.cz (K.C.); petr.konrad@fsv.cvut.cz (P.K.); premysl.kheml@fsv.cvut.cz (P.K.); maramich@cvut.cz (M.M.); jindrich.fornusek@fsv.cvut.cz (J.F.); petr.konvalinka@fsv.cvut.cz (P.K.); 2 Department of Electrotechnology, Faculty of Electrical Engineering, Czech Technical University in Prague, Technická 2, 166 27 Prague, Czech Republic; kuenzel@fel.cvut.cz

**Keywords:** concrete manufacturing, efficient manufacturing, prefabrication, 130 MPa, quality factor, non-destructive testing, flexural strength

## Abstract

Steel fiber-reinforced concrete (SFRC) can exhibit markedly improved mechanical performance when the fibers are preferentially aligned along the principal tensile stress directions. One method of aligning steel fibers is using magnetic methods. However, most existing magnetic alignment techniques rely on solenoids and are restricted to one-dimensional alignment and relatively small specimen sizes. This paper presents a novel planar magnetic orientation device capable of producing arbitrary two-dimensional fiber layouts and demonstrates its applicability from laboratory-scale proof-of-concept tests to large high-performance fiber-reinforced concrete (HPFRC) structural elements. The concept is first verified on transparent ultrasound gel specimens, where image analysis confirms fiber orientation in the prescribed angles. The method is then applied to small prismatic HPFRC specimens (40 mm × 40 mm × 160 mm) with fiber contents of 0.5%, 1.0%, and 1.5%, exposed to different magnetic field intensities (80 mT–140 mT). Flexural tests show increases in average flexural strength compared to non-oriented reference specimens, with 100 mT providing the most efficient alignment for the investigated mixture. A non-destructive electromagnetic method based on the measurement of the quality factor *Q* of a coil correlates well with flexural strength. Finally, the device is integrated with an industrial robot and used to orient fibers in large HPFRC slabs (1000 mm × 410 mm), achieving an average increase in flexural tensile strength of about 64% relative to non-oriented slabs. The results demonstrate that planar magnetic orientation is a promising approach for tailoring fiber layouts in SFRC structural elements and for enabling automated, programmable manufacturing.

## 1. Introduction

Steel fiber-reinforced concrete (SFRC) has been extensively studied for its enhanced mechanical performance, particularly in terms of crack resistance, toughness, and tensile and flexural strength [1,2,3,4,5]. However, the effectiveness of fiber reinforcement strongly depends not only on the volume and geometry of the fibers but also on the orientation and distribution of the fibers within the cementitious matrix [6,7,8,9]. Random orientation, which is typical in conventional casting processes, often leads to suboptimal performance, especially in structural elements subjected to tensile stress [9,10].

To address this, researchers have explored various techniques to control fiber orientation, including gravitational alignment, casting flow control, mechanical vibration, and magnetic or electric fields [11,12,13]. Among these, magnetic alignment using electromagnetic coils has shown promising results in directing ferromagnetic fibers along specific axes, significantly improving the mechanical properties of SFRC under bending and tension [14,15,16,17,18,19].

The common approach involves passing fresh cementitious material through a magnetic coil, aligning the fibers parallel to the magnetic field lines. While effective, this method poses significant limitations. The alignment is typically restricted to one direction, and the size of specimens, particularly their cross-sectional dimensions, is constrained by the geometry of the magnetic coil [15,20]. Moreover, the method is not easily scalable or adaptable to complex structural geometries where multidirectional fiber alignment may be required. The coil devices especially cannot be used for slab elements with dimensions approaching realistically manufactured (prefabricated) elements.

To overcome the limitations of more traditional coil-based alignment, an innovative orientation device was developed, capable of aligning fibers in any direction within a two-dimensional plane. This device would use combinations of permanent magnets to create a magnetic field through the thickness of slab elements. That will allow the orientation of steel fibers along specific paths, similarly to how traditional reinforcing bars are placed in concrete. Such orientation procedure is primarily aimed at the prefabrication industry and (ultra) high-performance fiber-reinforced concrete (HPFRC) where thicknesses of the slabs will be low compared to ordinary reinforced concrete (thin-walled elements), which will alow the through-thickness orientation. The specific concrete elements include protective barriers, permanent formwork, thin bridge structural elements, ceiling or facade panels etc.

This study as structured as follows. Initial experiments were conducted using an ultrasound gel matrix continued with HPFRC, with a custom-built prototype that generates a controlled magnetic field between two permanent magnets, enabling local alignment of steel fibers along the desired paths. In prospective industrial applications, this device—or the mold containing the fresh concrete—can be mounted on a robotic arm, allowing programmable movement in a 3D space and full automation of the orientation process. Key parameters affecting the alignment process include magnetic field strength and fiber content, which are investigated in this research.

The primary advantage of this method lies in its ability to create a specific fiber orientation in structural elements. By aligning fibers according to anticipated stress paths, particularly those defined by the strut-and-tie method, it is possible to improve resistance to various failure modes such as bending, splitting, punching, and shear. This study presents the initial experimental results, validates the proposed orientation method.

## 2. Experimental Program

The experimental program was designed to systematically evaluate the functionality and applicability of the proposed fiber orientation principle, progressing from simplified surrogate materials to realistic cementitious composites and automated processes. The program is divided into three stages: (i) verification of principle functionality using an ultrasound gel, which provides transparency for direct observation of fiber alignment; (ii) validation on small prismatic HPFRC specimens (40 mm × 40 mm × 160 mm), enabling mechanical testing to assess improvements in flexural performance; and (iii) demonstration of automated fiber alignment in large slab specimens using an industrial robot, which illustrates the potential for scalable and programmable application for manufacturing of structural elements.

### 2.1. Material

The material used in this study was an HPFRC made of common high-performance concrete constituents. The mixture is given in Table 1. The manufacturer of the silica fume (Elkem, Oslo, Norway) specifies its specific surface area between 15 m^2^/g and 35 m^2^/g, with primary particles size of about 0.15 μm. The manufacturer of the silica flour (Sklopísek Střeleč, Újezd pod Troskami, Czech Republic) specifies its specific surface area as 3.76 m^2^/g, with average particles size of 16 μm. The optimization of sand particle distribution was addressed in our previous research [21]. It was important for the magnetic orientation for the mixture to achieve certain rheological properties, so fibers would be allowed to turn in the magnetic field, but at the same time sinking of fibers was prevented. The rheological suitability of the matrix for magnetic fiber orientation has been verified in several previous studies. A detailed rheological characterization of a comparable cementitious matrix, including yield stress and flow-curve measurements using a rotational rheometer and Herschel–Bulkley modeling, was performed in [22]. These measurements established the rheological range necessary for fibers to rotate without excessive sinking. Furthermore, the ability of mixtures with these rheological properties to enable effective magnetic orientation of steel fibers was experimentally demonstrated in [23]. In these studies, the mixture exhibited the same functional behavior as used in the present work—allowing fiber rotation under magnetic torque while preventing sedimentation. Therefore, in this manuscript we report the mini-cone flow test primarily as a practical indicator confirming that the mixture meets the rheological window already defined and validated in the above studies. The mixture achieved 280 mm diameter flow in a mortar mini cone test (100 mm lower diameter, 70 mm upper diameter, 60 mm height of the cone) without dropping or agitating the table. Fibers were Weidacon FM (Stahl-und Fasertechnik, Hemer, Germany) high-strength steel, length 13 mm, diameter 0.15 mm, aspect ratio 87.

### 2.2. Verification of Principle Functionality on the Ultrasound Gel Specimens

**Figure 1:**
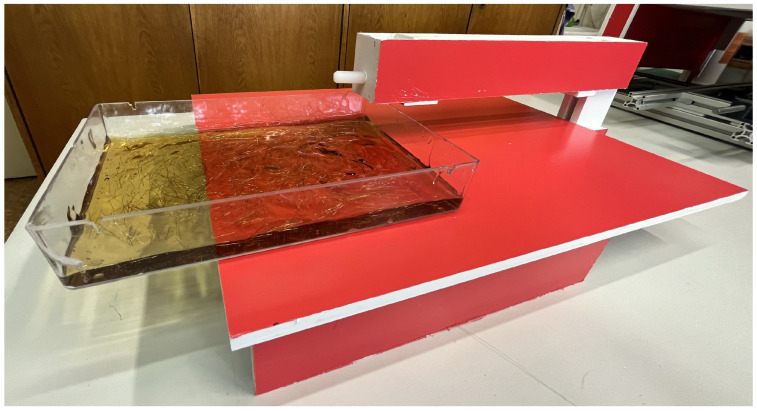
Device for fiber orientation inside the ultrasound gel.

To verify the feasibility of the proposed fiber orientation procedure, preliminary experiments were carried out using a transparent ultrasound gel matrix. The ultrasound gel was selected as a surrogate material for fresh cementitious composites because of its similar rheological properties [22] and, importantly, its optical transparency, which allows direct observation and quantitative evaluation of fiber orientation. Therefore, the main motivation of this test was not to create a perfect equivalent to the specific concrete mixture, but to asses the feasibility of the basic magnetic orientation setup—the magnetic field parameters combined with the steel fibers and two-dimensional movement of the material inside the magnetic field. Fibers were uniformly mixed into the gel and cast into a transparent plastic box with dimensions of 180 mm × 260 mm × 20 mm. The orientation device consisted of a pair of three neodymium magnets (30 mm × 30 mm × 7.5 mm) arranged at the top and bottom of the specimen, with a spacing of 65 mm between them. The magnets were held in place using a structure made of foamed PVC, which is in Figure 1. The configuration produced a magnetic induction of 34 mT in the alignment zone, as measured using a F.W. Bell 5180 Tesla meter (Parker Meggit, Coventry, UK). This magnetic induction was chosen, as it was discovered in the authors’ previous research [24], that the ultrasound gel requires approximately one third magnetic field strength compared to concrete.

Specimens were manually passed through the alignment zone in different directions to investigate the capacity of the system to generate uniform and multidirectional orientation. Three principal configurations were tested—horizontal alignment (fibers aligned along the x-axis), vertical alignment (perpendicular to the x-axis) and cross alignment (fibers oriented diagonally). Before and after magnetic treatment, the fiber orientation was recorded using top-view photographs. Image analysis was performed with FIJI ImageJ software (version 1.54) and the OrientationJ plugin (version 2.0.7) to objectively quantify orientation angles and distribution histograms.

### 2.3. Verification on Small Prismatic Specimens

In the second stage of the experimental program, the orientation device was applied to prismatic mortar specimens with dimensions of 40 mm × 40 mm × 160 mm. These specimens were prepared with varying steel fiber volume fractions of 0.5%, 1.0%, and 1.5% in order to investigate the influence of fiber content on alignment efficiency and mechanical performance. For each fiber dosage, a total of 20 specimens were cast. Each series was subdivided into five sets of four specimens: one reference set without exposure to a magnetic field, and four sets exposed to magnetic field intensities of 80 mT, 100 mT, 120 mT and 140 mT, respectively. Fiber orientation was induced by passing the fresh specimens through a planar magnetic field generated by the orientation device at an approximate speed of 3 cm/s.

**Figure 2:**
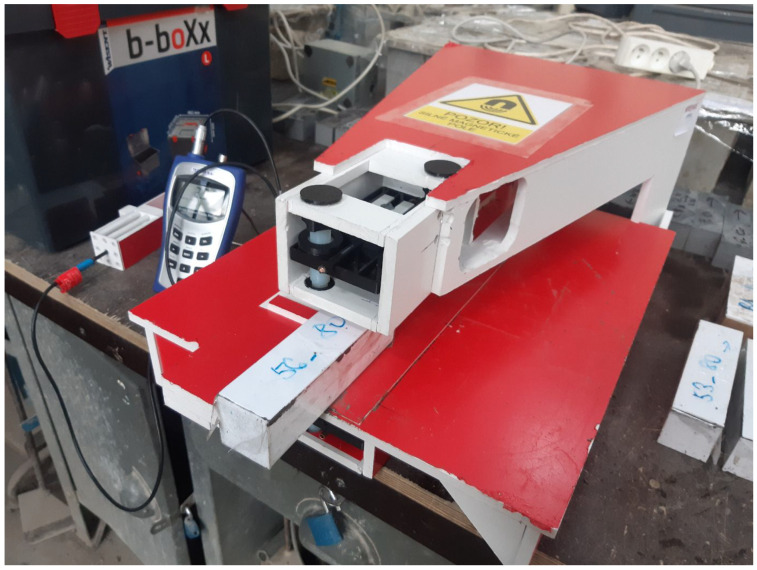
The whole device for fiber orientation.

Once the mould was filled with fresh material, it was sealed with a top cover to prevent leakage during the magnetic alignment process. Figure 2 illustrates the magnetic orientation setup with the closed mould in position. The main components of the device were fabricated from foam PVC and 3D-printed plastic parts in order to minimize interference with the magnetic field. The system consisted of a base table supporting the filled mould and a cantilevered arm positioned above it. One set of permanent magnets was mounted at the tip of the arm, while the second set was located directly below it in the table. Each magnetic set comprised of four NdFeB magnets (60 mm × 30 mm × 15 mm, grade N42, magnetic pull 56 kg, Unimagnet, Praha, Czech Republic) arranged with opposite poles facing each other across their largest faces. Together, the assembly formed a magnetic block measuring 60 mm × 30 mm × 60 mm, creating an effective orientation zone approximately 50 mm wide.

**Figure 3:**
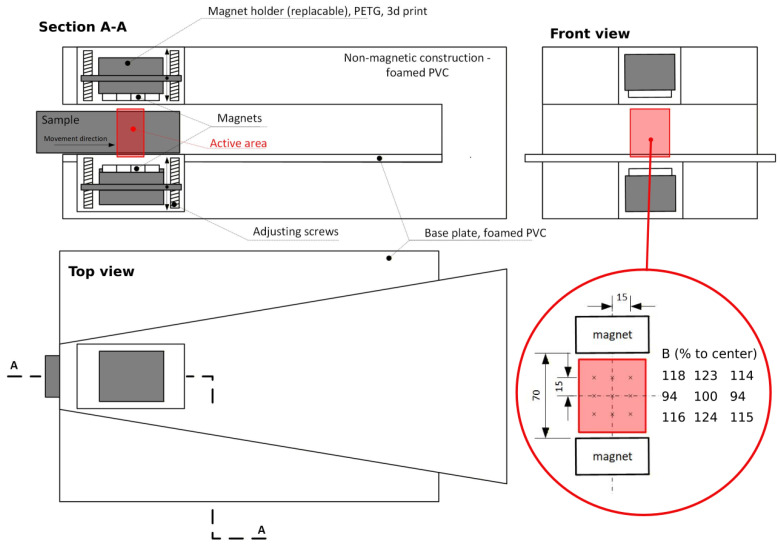
Schematic of the neodymium magnets.

Detailed schematic of the orientation device is in Figure 3, showing its geometry and magnet position. The detail of the magnet active area (in red) shows an example of measuring the magnetic field intensity *B* using the aforementioned Tesla meter. Probe of the meter was placed inside a plastic fixture with nine holes, to hold the probe in precise positions, as indicated by the schematic. Magnetic field intensity was changed by moving the set of magnets using the adjusting screws. The nominal value (80 mT, 100 mT, 120 mT and 140 mT) was set to be in the center of the space. The field intensity was then higher closer to the magnets and slightly lower in the middle plane to the sides of the active area. The goal was to achieve symmetrical intensities. The schematic in Figure 3 shows this field variation as percentages of the nominal central value for a case, when the magnets were 70 mm apart. Moving the magnets changes the field’s shape, but not significantly in this range of nominal intensities.

**Figure 4:**
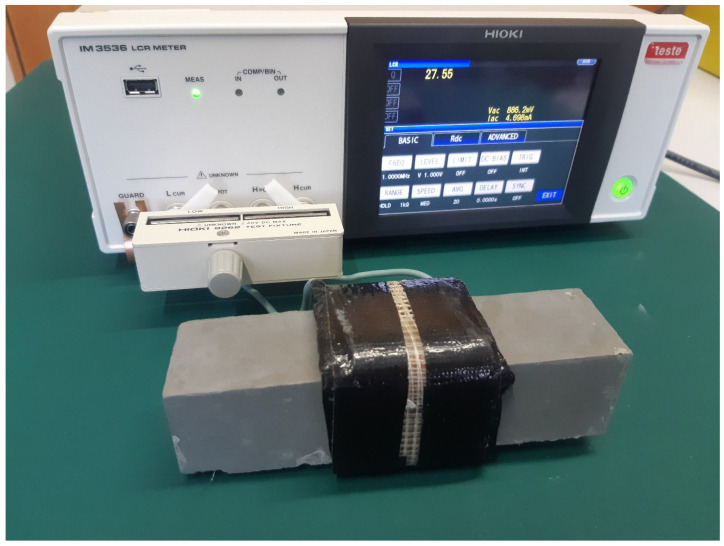
The non-destructive measurement set up.

After demoulding, the specimens were subjected to non-destructive testing at three positions along their length: at the beginning, middle, and end of the prism. Here, the “beginning” refers to the section that entered the magnetic field first during the alignment process. These measurements provided insight into the consistency of fiber orientation along the specimen length and the potential effects of field gradient or flow during casting. The measurement procedure was carried out as follows: each specimen was inserted into the measuring coil, which was connected to a HIOKI 3536 LCR impedance meter (HIOKI, Nagano, Japan), as illustrated in Figure 4.

The quality factor (Q) was recorded using this setup. The measured Q value strongly depends on the coil core material, which was either air (for reference measurements) or the concrete specimen itself. The measuring coil was made of copper wire with a diameter of 1.8 mm and consisted of 28 turns. Measurements were conducted over a frequency range from 1 MHz to 3 MHz, and the reported results correspond to the peak Q values observed within this range [25]. Subsequently, all specimens were tested destructively under three-point bending (100 mm total span) in order to evaluate the influence of fiber alignment on flexural behavior.

### 2.4. Automated Slab Specimens Produced with an Industrial Robot

In the third stage, the fiber orientation device was integrated with an industrial robot (KR 16 R1610-2, KUKA, Augsburg, Germany) to demonstrate the feasibility of automated large-scale fiber alignment. Four slab specimens with dimensions of 1000 mm × 410 mm × 50 mm were produced. Two specimens served as references and were cast without magnetic field exposure, while the remaining two were subjected to magnetic orientation.

**Figure 5:**
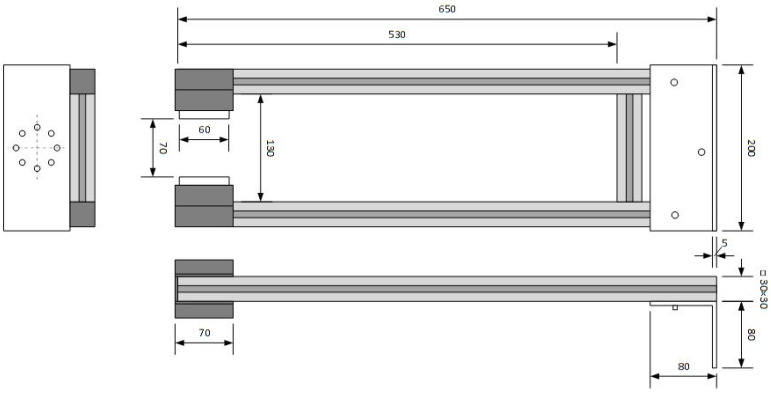
A custom-built magnetic tool equipped with permanent magnets.

The fresh concrete was cast into plastic moulds without mechanical vibration in order to minimize fiber segregation. After casting, each mould was placed on a fixed platform adjacent to the robotic arm. A custom-built magnetic head equipped with permanent magnets (Figure 5—the magnets are the same as for the prismatic specimens in Figure 3) was mounted on the robotic arm to generate a controlled magnetic field. The custom-built magnetic tool consisted of a frame made of aluminium profiles and two sets of permanent neodymium magnets housed in plastic casings. Each magnetic set comprised of eight NdFeB magnets (dimensions 60 × 30 × 15 mm, grade N42, magnetic pull 56 kg, Unimagnet, Praha, Czech Republic), arranged in a 2 × 4 configuration with alternating polarity. The two magnet assemblies were positioned opposite each other to form an effective magnetic channel capable of generating 100 mT magnetic field across the specimen thickness. The frame was designed for rigidity while maintaining low weight, enabling precise positioning of the magnetic head by the robotic arm.

**Figure 6:**
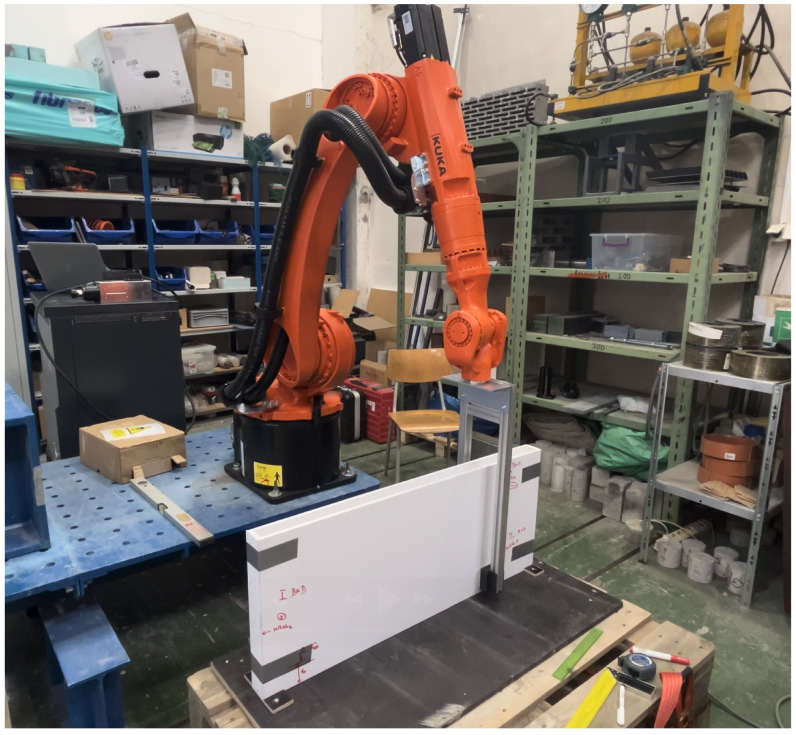
Orientation procedure with the industrial robot and a filled mould.

**Figure 7:**
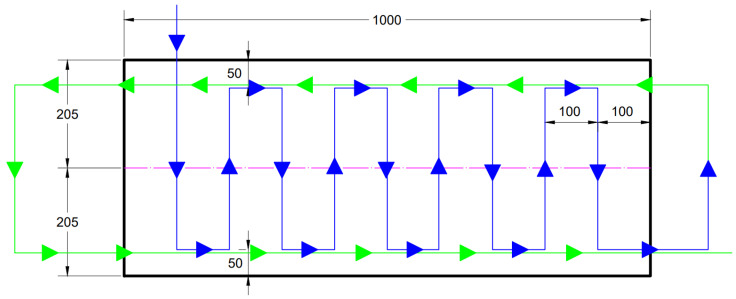
The HPFRC slab with the magnet path highlighted. Blue is part of the path orienting the vertical fiber channels, green is the path’s continuation for the horizontal channels. The central purple line shows the subsequent cutting of the specimen.

The robot was programmed to move around the mould at a constant speed of 3 cm/s, creating a defined sequence of fiber orientations (Figure 6). First, fibers were oriented vertically to simulate shear reinforcement (analogous to stirrups), subsequently in the same specimen, fibers were oriented horizontally at both the upper and lower surfaces to enhance flexural strength. A schematic illustration of the intended fiber layout is provided in Figure 7. After 28 days of curing, the slabs were cut longitudinally into two halves and tested in four-point bending, ensuring that the horizontally oriented fibers were located towards the tensile (bottom) surface. The upper half was, therefore, turned upside down, so both halves were tested with the strengthened side on the bottom. The reference specimens underwent identical cutting and testing procedures.

**Figure 8:**
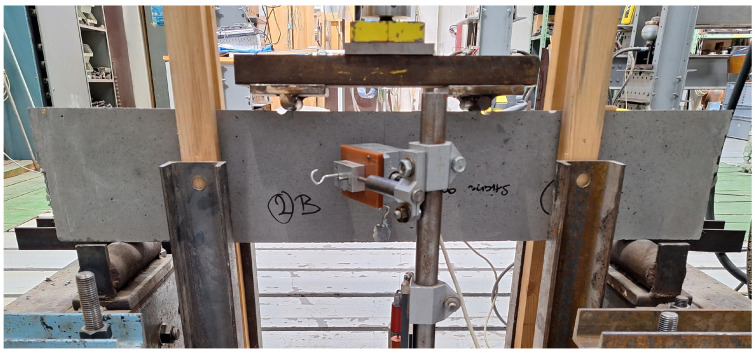
Four-point bending test.

The destructive tests were carried out under four-point bending configuration (800 mm total span, 300 mm center span), as shown in Figure 8. Due to the slender geometry of the specimens and concerns regarding potential lateral instability during loading, both sides of each specimen were equipped with auxiliary rails to prevent lateral torsional buckling. The load was applied through two symmetrically positioned rollers to ensure a constant bending moment in the central region. The specimens were tested under displacement control at a loading rate of 0.5 mm/min until the applied force decreased to zero, indicating complete failure.

## 3. Results and Discussion

### 3.1. The Ultrasound Gel Experiments

**Figure 9:**
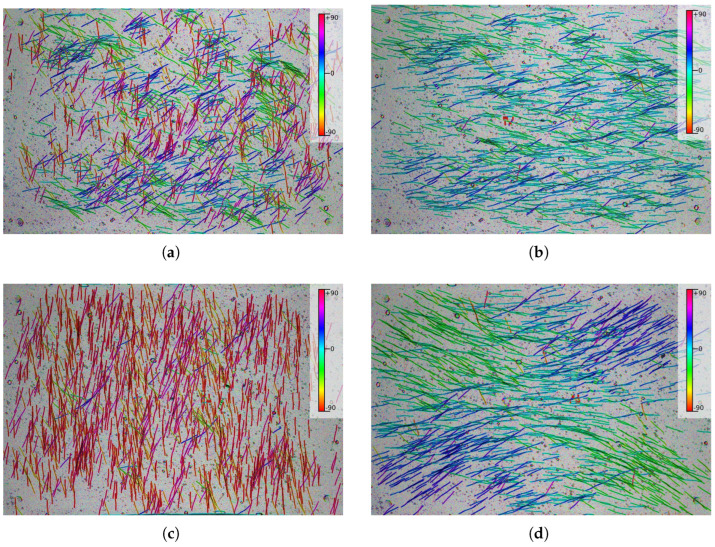
Image analysis of fibers oriented in the ultrasound gel experiments (**a**) before orientation (random) (**b**) horizontal (**c**) vertical (**d**) cross orientations. Colors represent the fiber angle.

**Figure 10:**
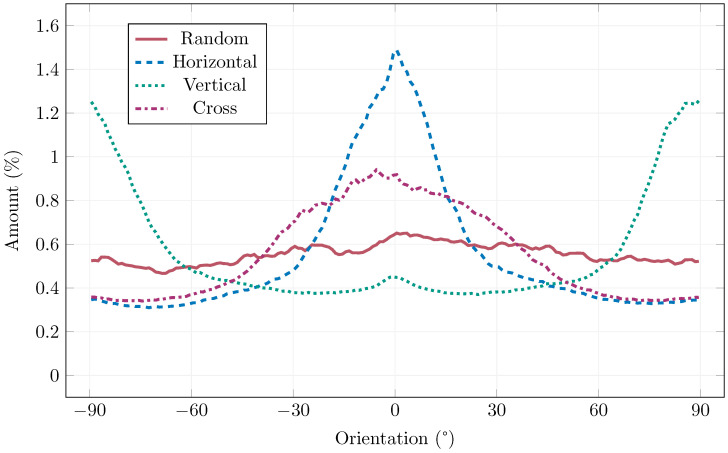
Results of the image analysis of the ultrasound gel experiments.

The image analysis objectively confirmed that the fibers were successfully aligned in all predefined directions. Figure 9 shows images of the sample after analysis, showing clearly the alignment of fibers. A summary of the orientation results for all configurations is shown in Figure 10. In the graph, the horizontal axis represents the fiber orientation angle, while the vertical axis shows the relative fiber volume percentage for each angle. Distinct peaks can be observed near 0° for the horizontally oriented samples and around 90° for the vertically oriented ones, confirming the targeted alignment directions. Conversely, the reference samples with randomly distributed fibers exhibit a nearly uniform distribution curve, indicating no preferential orientation. These results clearly demonstrate that the magnetic field effectively directs the ferromagnetic fibers along the desired paths, while the degree of alignment corresponds to the magnetic field orientation applied during the process. At the same time, it can be observed from the images, that fibers were not dragged behind the magnet to form chains and be displaced from their original positions, i.e., only orientation, not spacial distribution changed.

### 3.2. Small Prismatic Specimens

**Figure 11:**
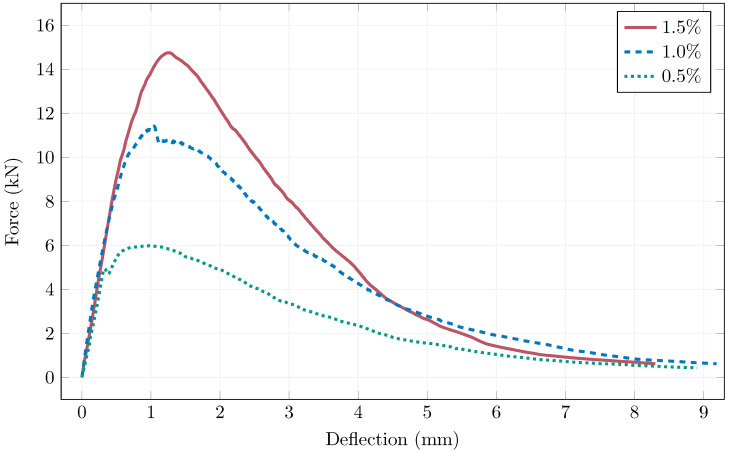
Examples of load-displacement diagrams of the small prismatic specimens.

Figure 11 shows selected examples of load-displacement diagrams of the three fiber percentages, as the rest of the diagrams exhibited practically identical shapes. There can be seen a small region of deflection hardening before reaching the peak force, followed by a pronounced deflection softening, as expected from a fiber-reinforced concrete. The 0.5% example exhibits the shortest deflection hardening, where the linear region reaches almost to the point of maximum force, indicating that 0.5% fiber volume is not fully utilizing the material’s potential. On the other hand, it can be seen that the peak load difference between 1.0% and 1.5% is not as large compared to between 0.5% and 1.0% pointing to reaching certain level, where further increase in fiber dosage would return diminished results (for this specific matrix design). Also, the magnetic orientation process must be taken into consideration, where too dense fiber volume cannot be oriented properly.

**Figure 12:**
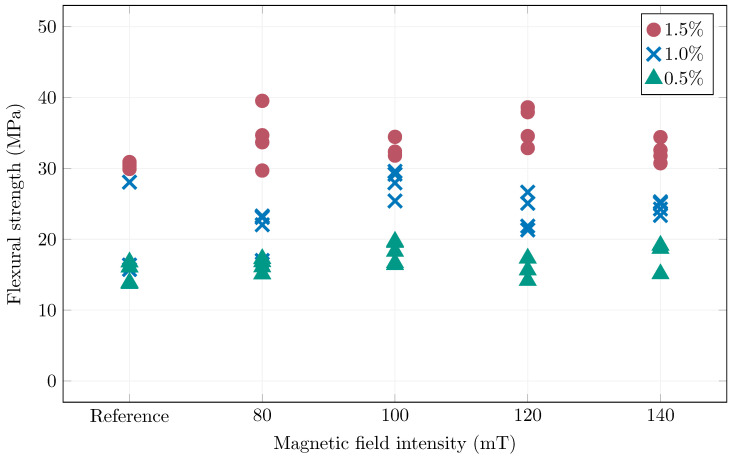
Summary of the achieved flexural strengths.

Table 2 summarizes the flexural strengths obtained from testing all of the small prismatic specimens. Figure 12 then graphically shows these results related to the manufacturing method, i.e., the magnetic field intensity (reference is without magnetic orientation). The 0.5% and 1.0% fiber specimens exhibited the best results when oriented by 100 mT magnetic field intensity. For the 1.5%, this intensity also resulted in good mechanical performance, but higher spread, pointing to 120 mT as better. Overall it can be said the magnetic orientation was achieved sufficiently by 100 mT and higher intensities were wasteful. However it must be noted, that this cannot be a general recommendation for magnetic orientation, as the orientation process must be matched to the specific concrete matrix (its rheology) and fibers.

The non-destructive measurements of the specimens are summarized in Table 3. The testing was carried out at three longitudinal positions along each prism—at the beginning, middle, and end. The term beginning refers to the portion of the specimen that entered the magnetic field first during the alignment process, while the end denotes the section that exited the field last. The measured quality factor reflects the electromagnetic response of the specimen; a lower Q value corresponds to a higher degree of fiber alignment in the direction of the measuring coil’s axis or, alternatively, to a lower local fiber volume fraction.

From the results, a consistent trend can be observed. Q values recorded in the middle sections of the specimens are generally lower than those measured at the beginning or the end. This indicates that fibers in the central region are either more effectively oriented or more densely concentrated. In contrast, no significant difference is apparent between the beginning and end positions, suggesting that the alignment process remains uniform along the direction of specimen movement through the magnetic field. It also confirms that fibers are not dragged behind the magnet. This indicates that the fibers are likely oriented with similar success along the length of the specimen, but there are fewer fibers in the central region because the mix was placed into the mould in the middle and, due to flow during casting, the fibers were transported towards the mould boundaries.

The lower Q values in the central portion of the prisms are most likely caused by a wall effect—fibers located near the boundaries experience greater resistance to reorientation due to friction and limited space, whereas fibers in the middle of the specimen are freer to rotate and align under the influence of the magnetic field. This effect is more pronounced in specimens with higher fiber volume fractions, where mutual fiber interactions further restrict movement near the mould walls.

**Figure 13:**
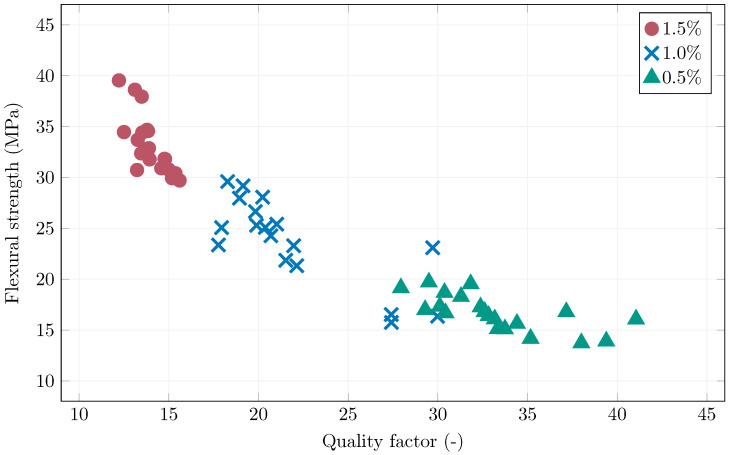
The quality factor and flexural strength correlation of the prismatic specimens.

Figure 13 shows the relationship between the measured quality factor in the center of the specimens and their achieved flexural strengths. Higher quality factor values always indicate lower influence of steel fibers, as presence of ferromagnetic material inside the coil will worsen it’s quality factor. Better oriented fibers (in the direction of the coil’s axis—parallel to the specimen’s length) have stronger influence on the coil. Therefore, the results are as expected as a clear trend can be seen with a correlation coefficient of −0.79. The sensitivity of the measuring coil was the highest for 0.5% fiber volume, and the lowest for 1.5% fiber volume. The quality factor analysis was able to spot problems with several specimens with the 1.0% fiber content, which indeed achieved subpar mechanical performance. Four 1.0% specimens exhibited quality factor larger than 25, two were references, where it would indicate undesirable fiber orientation as a result of fresh concrete placement. The other two were oriented by the magnetic field, however, the quality factor values obtained from either end of the specimen showed significantly different values (standard deviation of 2.3 and 3.9, as opposed to an average of the standard deviation across all specimens of 1.0), pointing to a possible problem with fiber concentration, where the volume of fibers responsible for the flexural strength in the center was locally lower.

### 3.3. Large Slab Specimens

**Figure 14:**
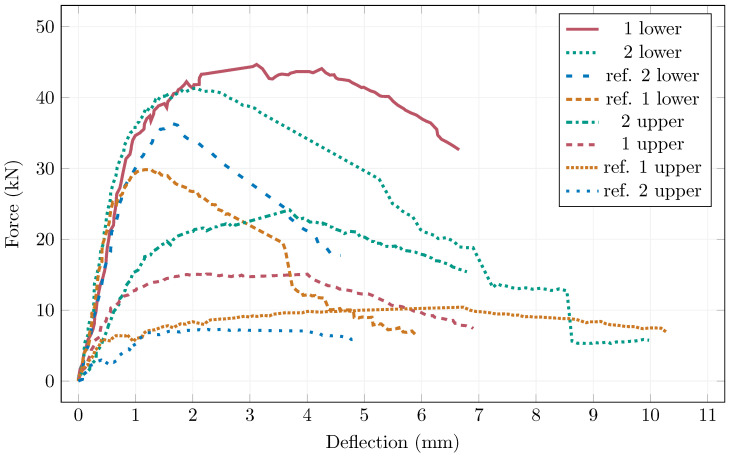
Load-displacement diagrams of the large slabs.

Table 4 shows the flexural tensile strengths of the halves of the slab specimens and Figure 14 shows the load-displacement diagrams. The flexural tensile strength results showed an average increase of approximately 64% for magnetically oriented specimens compared to the reference ones. When comparing the upper and lower halves of the slabs separately, the average percentage improvement between the reference and oriented specimens reached 37% for the lower halves and as much as 119% for the upper halves. A noticeable difference in flexural performance was observed between specimens taken from the upper and lower halves of the slabs—approximately 30% for the reference specimens and 47% for the magnetically oriented ones. This variation reflects the effect of fibre sedimentation during casting, where fibres tend to accumulate in the lower part of the element, resulting in higher flexural strength in that region. Despite the use of an optimised HPFRC mix, these findings indicate that partial fibre settlement still occurred under the specific laboratory conditions and larger specimen dimensions. The results therefore confirm that the magnetic orientation process effectively enhanced fibre alignment and mechanical performance, but also highlight the need for further rheological optimisation to maintain uniform fibre distribution in large-scale applications.

## 4. Conclusions

This study introduced and experimentally validated a novel planar magnetic orientation method for tailoring the steel fiber layout in cementitious composites. The research progressed from transparent surrogate material to small prismatic specimens and, finally, to large HPFRC slabs produced with an industrial robot. Based on the obtained results, the following conclusions can be drawn:

- The proposed planar magnetic device, using pairs of permanent neodymium magnets, is capable of generating well-defined fiber orientations in a transparent ultrasound gel as per the image analysis.
- When applied to small HPFRC prisms (40 mm × 40 mm × 160 mm) with fiber volume fractions of 0.5%, 1.0%, and 1.5%, the magnetic orientation improved flexural performance by 19.9%, 39.9% and 6.9%, respectively for 100 mT magnetic field intensity. For the studied mixture, field intensities of 100 mT and 120 mT were sufficient to achieve effective alignment.
- Non-destructive measurements based on the quality factor *Q* of a coil provided a sensitive indicator of fiber presence and alignment. Lower *Q* values corresponded to higher flexural strengths and better alignment, and a clear trend between *Q* measured in the prism center and the achieved flexural strength was observed.
- Integration of the magnetic orientation device with an industrial robot is feasible. For the HPFRC slabs (1000 mm × 410 mm × 50 mm), magnetically oriented specimens achieved on average about 64% higher flexural tensile strength than non-oriented references.
- Despite the use of an optimized HPFRC mixture, the large slabs showed noticeable differences between upper and lower halves, caused by fiber sedimentation in the fresh state. These findings highlight that, for large elements, the rheology of the concrete mix must be further tuned specifically for magnetic orientation.

Overall, the results confirm that planar magnetic orientation using permanent magnets is a viable and scalable method for controlling fiber alignment in HPFRC. In combination with non-destructive electromagnetic testing, it offers a powerful framework for both the manufacturing and quality control of advanced fiber-reinforced concrete elements. Future work will focus on optimizing mix rheology for larger structural members, extending the method to three-dimensional stress paths, and integrating feedback from non-destructive measurements into closed-loop robotic control of the orientation process.

## Figures and Tables

**Table 1:** Concrete mixture used in the study.

Constituent	Weight in 1 m^3^	Rel. to Cement
	(kg)	(-)
Cement 42.5 R	693	1.00
Silica fume	76	0.11
Silica flour	194	0.28
Water	194	0.28
HRWR	39	0.06
Sand 0/4 mm	145	0.21
Sand 0.6/1.2 mm	298	0.43
Sand 0.3/0.8 mm	125	0.18
Sand 0.1/0.6 mm	651	0.94
Sum	2416	3.49

**Table 2:** Average flexural strengths of the small prismatic specimens.

Fiber Content	Magnetic Field	Flexural Strength	Increase
(%)	(mT)	(MPa) (St. Dev.)	Rel. to Ref. (%)
1.5	0 (reference)	30.50 (0.44)	-
	80	34.40 (4.04)	12.8
	100	32.61 (1.25)	6.9
	120	36.00 (2.74)	18.0
	140	32.38 (1.56)	6.2
1.0	0 (reference)	20.05 (6.94)	-
	80	20.39 (3.35)	1.7
	100	28.04 (1.88)	39.9
	120	23.73 (2.56)	18.4
	140	24.49 (0.88)	22.1
0.5	0 (reference)	15.12 (1.54)	-
	80	16.31 (0.94)	7.9
	100	18.12 (1.54)	19.9
	120	15.71 (1.58)	3.9
	140	17.65 (2.20)	16.7

**Table 3:** Results of non-destructive measurements.

Fiber Content	Magnetic Field	Q Factor Beginning	Q Factor Middle	Q Factor End
(%)	(mT)	(-)	(-)	(-)
1.5	0 (reference)	16.51	15.03	15.80
	80	15.02	13.72	15.10
	100	14.63	13.88	14.66
	120	14.72	13.58	14.49
	140	15.02	13.58	13.89
1.0	0 (reference)	26.90	25.16	27.03
	80	26.25	25.64	26.87
	100	20.53	19.35	20.36
	120	21.59	20.97	21.54
	140	20.25	19.07	20.03
0.5	0 (reference)	41.63	38.90	40.78
	80	35.03	32.98	33.70
	100	31.94	30.71	32.19
	120	34.02	33.13	33.85
	140	31.39	30.29	29.93

**Table 4:** Summary of flexural tensile strength of the large slabs.

Specimen	Flexural Tensile Strength [MPa]
ref. 1 upper	4.3
ref. 1 lower	11.3
ref. 2 upper	3.2
ref. 2 lower	13.8
1 upper	6.3
1 lower	18.8
2 upper	10.0
2 lower	15.6

## Data Availability

The original contributions presented in this study are included in the article. Further inquiries can be directed to the corresponding author.
